# Fascicles and the interfascicular matrix show adaptation for fatigue resistance in energy storing tendons

**DOI:** 10.1016/j.actbio.2016.06.012

**Published:** 2016-09-15

**Authors:** Chavaunne T. Thorpe, Graham P. Riley, Helen L. Birch, Peter D. Clegg, Hazel R.C. Screen

**Affiliations:** a Institute of Bioengineering, School of Engineering and Materials Science, Queen Mary University of London, Mile End Road, London E1 4NS, UK; b School of Biological Sciences, University of East Anglia, Norwich Research Park, Norwich NR4 7TJ, UK; c Institute of Orthopaedics and Musculoskeletal Science, University College London, Royal National Orthopaedic Hospital, Stanmore, HA7 4LP, UK; d Department of Musculoskeletal Biology, Institute of Ageing and Chronic Disease, University of Liverpool, Leahurst Campus, Neston CH64 7TE, UK

**Keywords:** Tendon, Fascicle, Interfascicular matrix, Mechanical testing, Fatigue resistance, Creep

## Abstract

Tendon is composed of rope-like fascicles, bound together by interfascicular matrix (IFM). Our previous work shows that the IFM is critical for tendon function, facilitating sliding between fascicles to allow tendons to stretch. This function is particularly important in energy storing tendons, which experience extremely high strains during exercise, and therefore require the capacity for considerable inter-fascicular sliding and recoil. This capacity is not required in positional tendons. Whilst we have previously described the quasi-static properties of the IFM, the fatigue resistance of the IFM in functionally distinct tendons remains unknown. We therefore tested the hypothesis that fascicles and IFM in the energy storing equine superficial digital flexor tendon (SDFT) are more fatigue resistant than those in the positional common digital extensor tendon (CDET). Fascicles and IFM from both tendon types were subjected to cyclic fatigue testing until failure, and mechanical properties were calculated. The results demonstrated that both fascicles and IFM from the energy storing SDFT were able to resist a greater number of cycles before failure than those from the positional CDET. Further, SDFT fascicles and IFM exhibited less hysteresis over the course of testing than their counterparts in the CDET. This is the first study to assess the fatigue resistance of the IFM, demonstrating that IFM has a functional role within tendon and contributes significantly to tendon mechanical properties. These data provide important advances into fully characterising tendon structure-function relationships.

**Statement of Significance:**

Understanding tendon-structure function relationships is crucial for the development of effective preventative measures and treatments for tendon injury. In this study, we demonstrate for the first time that the interfascicular matrix is able to withstand a high degree of cyclic loading, and is specialised for improved fatigue resistance in energy storing tendons. These findings highlight the importance of the interfascicular matrix in the function of energy storing tendons, and potentially provide new avenues for the development of treatments for tendon injury which specifically target the interfascicular matrix.

## Graphical abstract

**Figure:**
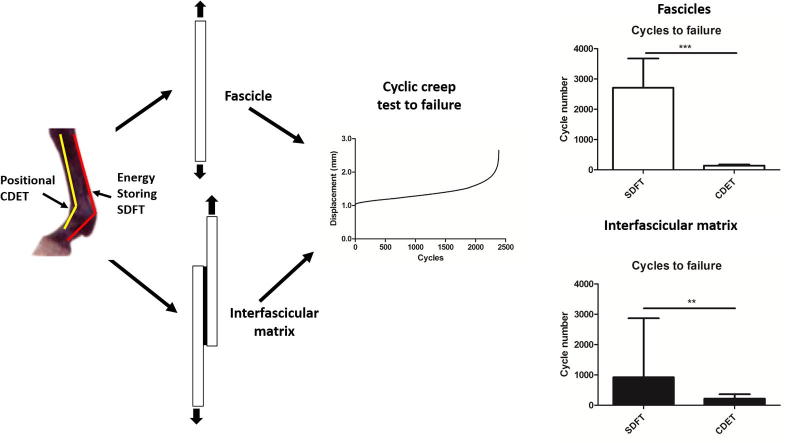

## 1 Introduction

Energy storing tendons, such as the human Achilles and patellar tendons, play an important role in locomotory efficiency, decreasing the energetic cost associated with movement [1], [2]. To enable this function, energy storing tendons have distinct mechanical properties, such as greater extensibility and elasticity leading to improved energy storage and return, when compared to tendons that are purely positional in function, such as the anterior tibialis tendon [1], [3], [4], [5]. Energy storing tendons also have superior fatigue resistance, withstanding a greater number of loading cycles prior to failure than positional tendons in mechanical tests using the whole tendon [6], [7].

Tendons are hierarchical fibre-composite materials, in which collagenous units are grouped together, forming subunits of increasing diameter [8]. At the higher hierarchical levels, the collagen is interspersed with a less fibrous, highly hydrated matrix, traditionally referred to as the ground substance [9]. The largest tendon subunit is the fascicle; with a diameter of approximately 300 μm, fascicles are visible to the naked eye and can be isolated by cutting longitudinally through the tendon. Fascicles are bound together by the interfascicular matrix (IFM), which is the largest hierarchical level of ground substance, and is also referred to as the endotenon. The IFM is rich in glycoproteins, elastin and collagens [9], [10], [11].

In order to fully understand tendon structure-function relationships, it is important to characterise the specialisations that result in enhanced energy storage in specific tendons. Our previous studies have demonstrated specialisation of both fascicles and IFM in energy storing tendons. The additional extensibility in energy storing tendons is provided by the IFM, which enables greater sliding between adjacent fascicles, resulting in higher levels of extension in the tendon as a whole [3]. In addition, both fascicles and the IFM are more elastic in energy storing tendons, demonstrating less hysteresis and stress relaxation during cyclic loading than in positional tendons [12], [13]. We have also shown that fascicles from energy storing tendons are more fatigue resistant than those from positional tendons, both in the bovine and equine model [13], [14], however no previous studies have assessed the fatigue resistance of the IFM and how this differs between tendons with differing functions.

In the current study, we adopted the equine model to assess the fatigue response of functionally distinct tendons. The horse is a relevant and accepted model for tendon research, as it is an athletic species which maximises energy efficiency by storage and release of elastic energy in the limb tendons. The predominant energy store in the horse is the forelimb superficial digital flexor tendon (SDFT), which has an analogous function to the Achilles tendon [15], [16], [17]. Indeed, tendon injuries in the SDFT show a very similar epidemiology, aetiology, and pathology to those seen in the human Achilles tendon [16], [17]. The anatomically opposing equine common digital extensor tendon (CDET) is an example of a positional tendon, functionally comparable to the human anterior tibialis tendon [18]. We tested the hypothesis that the IFM in the energy storing SDFT is more fatigue resistant than the IFM in the positional CDET, similar to the difference between the fascicles in the two tendon types.

## 2 Materials and methods

### 2.1 Sample collection and preparation

Forelimbs, distal to the carpus, were collected from horses aged 3–7 years (n = 4) euthanased at a commercial equine abattoir, as a by-product of the agricultural industry. Specifically, the Animal (Scientific Procedures) Act 1986, Schedule 2, does not define collection from these sources as scientific procedures. The SDFT and CDET were harvested from the forelimbs within 24 h of euthanasia. Whilst it was not possible to obtain a full exercise history for the horses, none of the tendons had clinical or macroscopic evidence of tendon injury. Tendons were wrapped in tissue paper dampened with phosphate buffered saline (PBS) and then in tin foil and stored at −80 °C. On the day of testing, tendons were thawed and fascicles, approximately 30 mm in length, were dissected from the mid-metacarpal region of the tendon as previously described (n = 6–8 per tendon) [19]. In addition, groups of two fascicles, bound together by IFM were also dissected from the same region (n = 6–8 per tendon) [3]. Fascicle hydration was maintained by storing the samples on tissue paper dampened with Dulbecco’s modified eagle medium (DMEM).

### 2.2 Determination of fascicle fatigue properties

Fascicle diameter was determined using a laser micrometer, measuring continuously along a 10 mm length in the central portion of the fascicle and taking the smallest diameter to calculate cross-sectional area, assuming a circular cross section [3]. Fascicles were secured in custom made individual loading chambers [20], with a grip to grip distance of 10 mm, and fascicle fatigue properties were determined using an Electroforce 5500 mechanical testing machine, equipped with a 22 N load cell (TA instruments, Delaware, USA), housed within a cell culture incubator (37 °C, 20% O_2_, 5% CO_2_). A pre-load of 0.1 N was applied to remove any slack within the samples. We have previously shown that fascicle failure strain is more consistent between samples than failure stress [3], Accordingly, one loading cycle to a displacement of 1 mm (10% strain, equivalent to 50% of predicted fascicle failure strain [19]) was applied to establish an appropriate and consistent peak load for cyclic fatigue testing. This peak load was subsequently applied to the fascicles in a cyclic manner at a frequency of 1 Hz until sample failure. Load and displacement data were recorded continuously throughout the test at a frequency of 100 Hz. In addition, the maximum and minimum load and displacement were recorded for each cycle.

### 2.3 Determination of IFM fatigue properties

Samples were prepared for IFM fatigue testing as previously described [3], [21]. Briefly, transverse cuts were made in the opposing ends of 2 fascicles bound together by IFM, leaving a consistent IFM length of 10 mm. The intact end of each fascicle was secured in the loading chambers and IFM fatigue properties were determined using an Electroforce 5500 mechanical testing machine, equipped with a 22 N load cell, housed within a cell culture incubator (37 °C, 20% O_2_, 5% CO_2_). A pre-load of 0.02 N was applied to remove any slack within the samples. IFM failure extension is more consistent between cycles than failure force [3], therefore one loading cycle of 1 mm displacement was applied, which is equivalent to 50% of the predicted failure extension [3], to find the peak load. This load was subsequently applied to the IFM in a cyclic manner at a frequency of 1 Hz until sample failure. Load and displacement data were recorded continuously throughout the test at a frequency of 100 Hz. In addition, the maximum and minimum load and displacement were recorded for each cycle.

### 2.4 Data analysis

**Fig. 1:**
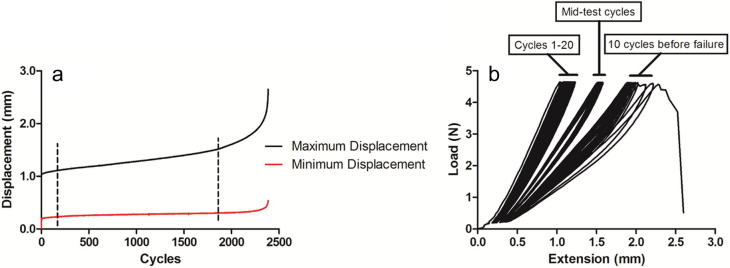
Example creep curves (a) showing the maximum and minimum displacement at each cycle during fatigue testing. The dotted lines indicate the linear region of the creep curve (secondary creep), the gradient of which was calculated. Example force extension curves (b); hysteresis was calculated over cycles 1–10 and 11–20, the middle 10 cycles of the test, and the 10 cycles immediately prior to failure. Maximum loading and unloading stiffness was calculated for cycle 1, 10, mid-test cycle, and 10 cycles and 1 cycle before failure.

For each test, the number of cycles to failure was recorded. The maximum and minimum displacement data were used to plot creep curves to failure (Fig. 1a) and the gradient of the maximum and minimum displacement curves during secondary creep were calculated.

The load and displacement data were used to plot force extension curves (Fig. 1b). Hysteresis over cycles 1–10, 11–20, the middle 10 cycles and the last 10 cycles prior to failure was calculated by dividing the area between the loading and unloading curves (energy dissipated) by the area under the loading portion of the curve (energy input), and expressed as a percentage. In addition, the maximum loading and unloading stiffness was calculated for cycle 1, cycle 10, the mid-test cycle, 10 cycles prior to failure and the last cycle prior to failure.

Fascicle elongation was calculated at cycle 10 and at the cycle prior to failure by subtracting the maximum extension at cycle 1 from the maximum extension in these cycles. It was not possible to calculate IFM elongation, relative to the first cycle, as the low forces involved in this load controlled experiment required several cycles to fully stabilise, therefore the elongation between cycle 10 and the cycle prior to failure was calculated.

### 2.5 Statistical analysis

Statistical differences between tendon types were determined using Analysis of Variance (Minitab 17). A general linear model was fitted to the data, with tendon type and horse number included as factors. Data were tested for normality using the Anderson–Darling test. Data that did not follow a normal distribution were transformed using a Box-Cox transformation. Data are displayed as mean ± SD. To assess correlations between initial mechanical parameters (hysteresis and elongation at cycle 10) and the number of cycles to failure, Spearman correlation coefficients were calculated.

## 3 Results

Fascicle and IFM fatigue properties are shown in Table 1.

### 3.1 Fascicle fatigue properties

Fascicles from the SDFT resisted significantly more loading cycles before failure than those from the CDET (p < 0.001).

**Fig. 2:**
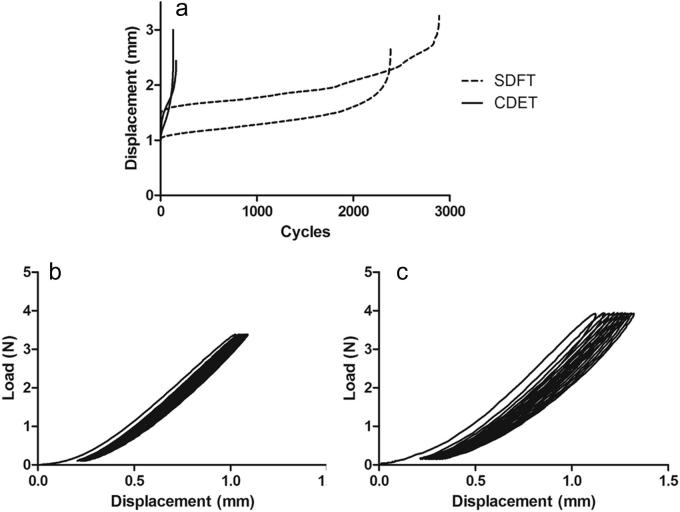
Typical creep curves for fascicles from the SDFT and CDET (a). Typical loading and unloading curves for cycles 1–10 of testing of SDFT (b) and CDET (c) fascicles.

**Fig. 3:**
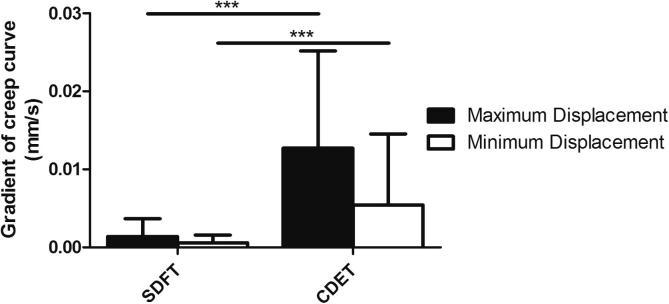
Gradient of the maximum and minimum creep curves of fascicles from the SDFT and CDET. Data are displayed as mean ± SD. ^***^p < 0.001.

Typical creep and force extension curves for fascicles are shown in Fig. 2. The gradient of the maximum and minimum creep curves were significantly greater in the CDET than in the SDFT (p < 0.001; Fig. 3).

**Fig. 4:**
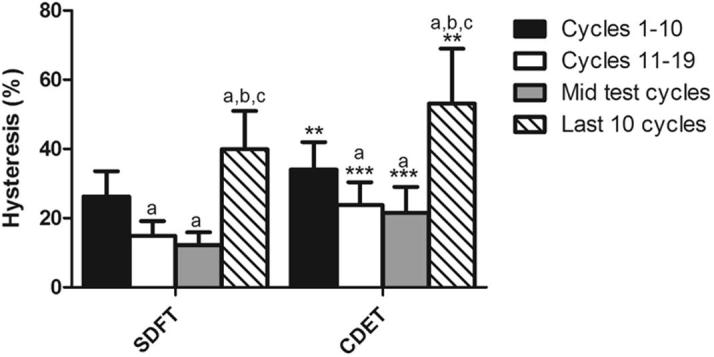
Hysteresis at different points throughout fatigue testing in fascicles from the SDFT and CDET. Data are displayed as mean ± SD. ^∗^Indicates significant difference between tendon types: ^**^p < 0.01; ^***^p < 0.001. ^a^Indicates significant difference relative to cycles 1–10 (p < 0.001); ^b^indicates significant difference relative to cycles 11–20 (p < 0.001); ^c^indicates significant difference relative to mid test cycles (p < 0.001).

Fascicle hysteresis was significantly greater in the CDET than in the SDFT at all time points that were assessed (p < 0.01). In both tendon types, hysteresis decreased significantly until the mid-test cycles, and then increased significantly in the final 10 cycles prior to failure (p < 0.001; Fig. 4).

**Fig. 5:**
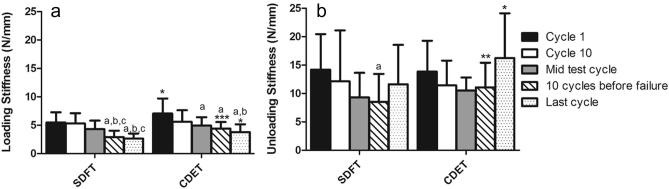
Loading stiffness (a), and unloading stiffness (b) in fascicles from the SDFT and CDET. Data are displayed as mean ± SD. ^∗^Indicates significant difference between tendon types: ^*^p < 0.05; ^**^p < 0.01. ^a^Indicates significant difference relative to cycle 1 (p < 0.01); ^b^indicates significant difference relative to cycle 10 (p < 0.01); ^c^indicates significant difference relative to mid test cycles (p < 0.001).

Loading stiffness was significantly greater in fascicles from the CDET than those from the SDFT at cycle 1, and at both 10 and 1 cycles prior to failure (p < 0.05; Fig. 5a). In fascicles from both tendon types, loading stiffness decreased over the course of fatigue testing, with significantly lower values towards the end of the test (cycles 10 and 1 prior to failure) than at the start (cycle 1) (p < 0.01; Fig. 5a). Unloading stiffness was significantly greater in CDET fascicles than in SDFT fascicles, 10 cycles and 1 cycle prior to failure (p < 0.05; Fig. 5b). In the SDFT, unloading stiffness continued to reduce right through the test and only increased in the last cycle prior to failure (p < 0.01). In the CDET, unloading stiffness did not alter significantly with cycle number.

**Fig. 6:**
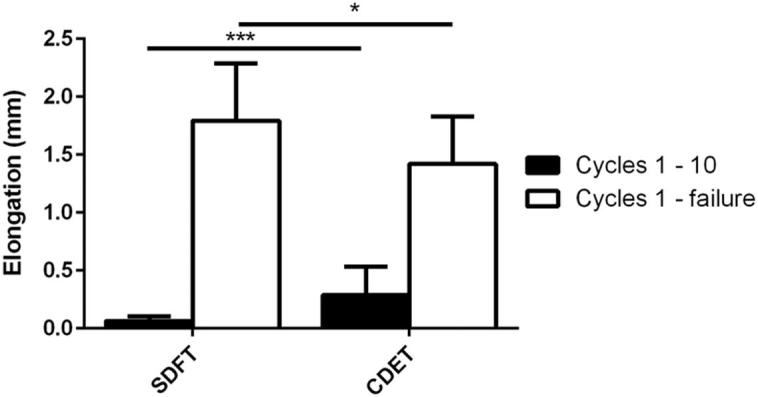
Fascicle elongation in the SDFT and CDET between the 1st and 10th cycle, and the 1st and final cycle. Data are displayed as mean ± SD. ^∗^Indicates significant difference between tendon types: ^*^p < 0.05; ^***^p < 0.001.

Initial fascicle elongation was greater in the CDET than in the SDFT (Fig. 6). However, by the last cycle prior to failure, the total fascicle elongation in the SDFT was greater than in the CDET (Fig. 6).

**Supplementary Fig. 1:**
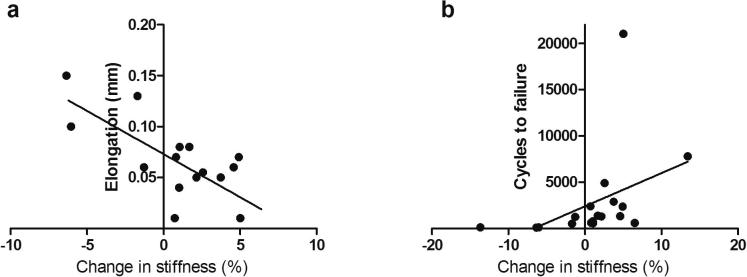
In the SDFT, change in loading stiffness from cycle 1 to cycle 10 showed a negative correlation with elongation over the same period (a; r = −0.61; p = 0.013), and was positively correlated with number of cycles to failure (b; r = 0.73; p = 0.006).

In fascicles from the SDFT, hysteresis over the first 10 cycles showed a significant positive correlation with elongation at cycle 10, and was negatively correlated with number of cycles to failure (Table 2). Elongation at the 10th cycle also showed a negative correlation with the number of cycles to failure (Table 2). The percentage change in maximum loading stiffness over the first 10 cycles showed a significant negative correlation with elongation, and was positively correlated with the number of cycles to failure (Table 2; Supplementary Fig. 1). There was no relationship between initial mechanical parameters and fatigue resistance in fascicles from the CDET.

### 3.2 IFM fatigue properties

The IFM in the SDFT was able to resist a significantly greater number of cycles to failure than the CDET IFM (p = 0.002).

**Fig. 7:**
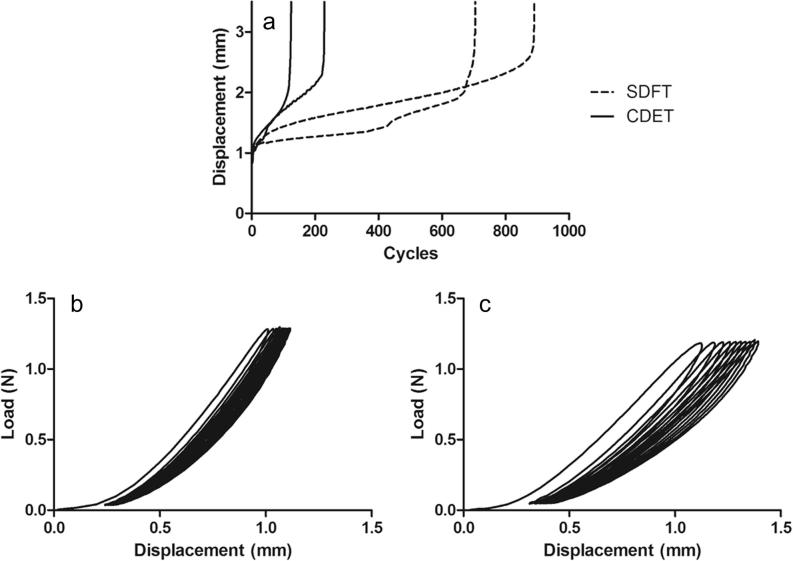
Typical IFM creep curves for samples from the SDFT and CDET (a). Typical loading and unloading curves for cycles 1–10 of testing of SDFT (b) and CDET (c) IFM samples.

**Fig. 8:**
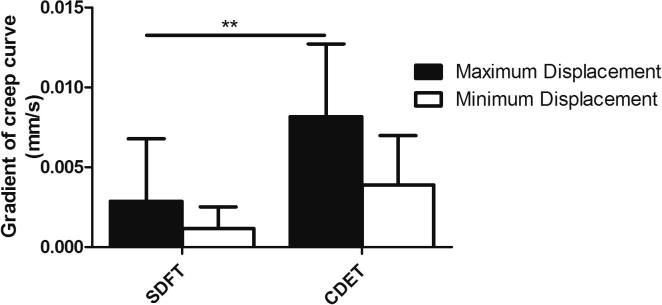
Gradient of the maximum and minimum creep curves of IFM from the SDFT and CDET. Data are displayed as mean ± SD. ^**^p < 0.01.

Typical creep and force extension curves for the IFM are shown in Fig. 7. The gradient of the maximum and minimum creep curves were significantly greater in the CDET IFM than in the SDFT IFM (p < 0.01; Fig. 8).

**Fig. 9:**
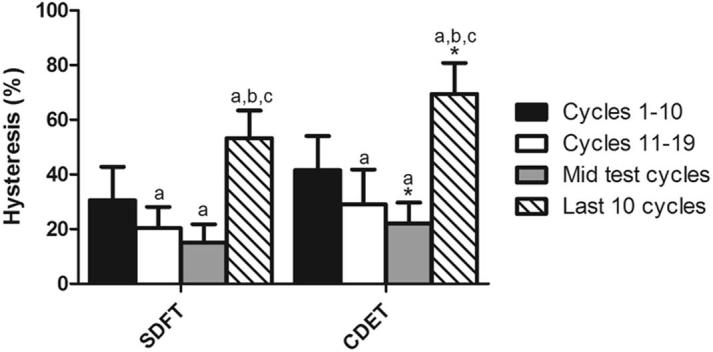
Hysteresis at different points throughout fatigue testing of IFM from the SDFT and CDET. Data are displayed as mean ± SD. ^∗^Indicates significant difference between tendon types: ^*^p < 0.05. ^a^Indicates significant difference relative to cycles 1–10 (p < 0.05); ^b^indicates significant difference relative to cycles 11–20 (p < 0.001); ^c^indicates significant difference relative to mid test cycles (p < 0.001).

There was a trend towards greater hysteresis in the CDET IFM than in the SDFT IFM throughout the test, which reached significance from the mid-test point onwards (p < 0.05; Fig. 9). Hysteresis varied over the course of the fatigue testing in a similar manner to that observed in fascicles, with a decrease until the mid-test cycles, followed by an increase in the 10 cycles prior to failure (Fig. 9).

**Fig. 10:**
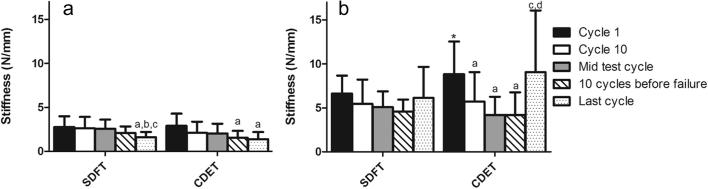
Loading stiffness (a), and unloading stiffness (b) in IFM from the SDFT and CDET. Data are displayed as mean ± SD. ^∗^Indicates significant difference between tendon types (p < 0.05). ^a^Indicates significant difference relative to cycle 1 (p < 0.01); ^b^indicates significant difference relative to cycle 10 (p < 0.05); ^c^indicates significant difference relative to mid test cycle (p < 0.05); ^d^indicates significant difference relative to 10 cycles before failure (p < 0.01).

Loading stiffness of the IFM did not differ between tendon types at any of the time points assessed. In both the SDFT and CDET, IFM loading stiffness decreased with increasing cycle number (Fig. 10a), and was significantly lower in the last cycle prior to failure (p < 0.05), just as seen in fascicles. IFM unloading stiffness was significantly greater in the CDET than in the SDFT at cycle 1 only (Fig. 10b; p < 0.05). In the SDFT, IFM unloading stiffness did not alter significantly with cycle number. In the CDET, IFM unloading stiffness decreased significantly after cycle 1, and then increased significantly in the last cycle prior to failure (Fig. 10b).

There was a trend towards greater IFM elongation between cycle 10 and the cycle prior to failure in the CDET than in the SDFT, but this was not significant (p = 0.1).

There was no relationship between initial mechanical parameters and number of cycles to failure in the IFM in either tendon type.

## 4 Discussion

Our previous studies have shown that the SDFT has lower levels of hysteresis and stress relaxation in both fascicles and IFM compared to the CDET during cyclic loading [12], suggesting that the SDFT may have superior fatigue properties. The current data support the hypothesis, demonstrating that both fascicles and IFM in the energy storing SDFT have a superior fatigue resistance when compared to those from the positional CDET.

There are several limitations to the current study that should be considered. It is evident that the data are highly variable, particularly with regard to the number of cycles to failure. Such variability is inherent to fatigue experiments, due to their sensitivity to any initial defect [22], and it is possible that some damage may have occurred to the samples during the dissection process. Samples were carefully observed and handled during both dissection and testing to minimise this, and the existence of statistical significance when comparing the fatigue properties of the two tendon types, despite the large variability, perhaps highlights the magnitude of difference in the properties assessed. The large variation in the results may also be due to variations in fascicle fatigue properties both within a tendon from one individual, and between individuals. When considering the IFM testing procedure, it is not possible to test IFM in isolation so there may be some contribution to the recorded mechanics from fascicles, however as IFM failure properties and stiffness are less than half that of fascicles, fascicle contribution to the measured IFM response is likely to be minimal. In addition, the unbalanced test design used for IFM testing may lead to some error associated with interface rotation and generation of tension perpendicular to the loading axis. However, it is not possible to use a balanced shear design without causing extensive damage to the samples during dissection.

Although the IFM is a looser matrix, it shows considerable fatigue resistance, particularly in the energy storing SDFT. It is not possible to directly compare fascicle and IFM tests due to different test designs used (uniaxial vs. shear), but it is still evident that IFM has significant capacity to resist fatigue loading in both tendon types, with hysteresis in the IFM only slightly greater than in fascicles. Indeed, the small lengths of IFM tested were able to resist loads of up to 2 N, and withstand many cycles prior to failure. This suggests that *in vivo*, where the IFM is continuous, it is able to resist significant loads and therefore manage sliding between fascicles which are likely to be discontinuous [23].

During IFM quasi-static tests to failure, we have previously demonstrated a significantly larger toe region in the SDFT, such that the extension and force at which the maximum stiffness is reached is significantly higher in the SDFT than in the CDET, demonstrating a greater capacity for interfascicular sliding at low forces in the SDFT [12]. However, in agreement with our previous findings [12], an analysis of the linear region of the force-extension curve shows does not identify any differences in maximum loading stiffness between the IFM in the SDFT and CDET. The interfascicular sliding facilitated by an elongated toe region in energy storing tendons enables them to withstand the high strains they experience [3], and recent studies suggest that the IFM in energy storing tendons has a specialised composition to enable this [10], [11]. It has been shown that the IFM in the energy storing SDFT is rich in elastin and lubricin as well as many proteoglycans and collagens [10], [11], providing both strength and elasticity. The IFM is also more abundant in the energy storing SDFT than in the positional CDET [21]. In addition, the IFM has a greater cellular content and a faster rate of turnover than the FM [10]. The shearing role of the IFM in energy storing tendons may predispose it to damage, therefore the faster rate of turnover in this region may be a mechanism by which damage to the IFM is preferentially repaired to maintain structural integrity.

When considering the fascicle response to fatigue loading, fascicles from the SDFT were able to resist almost 20 times more cycles to failure than those from the CDET, and exhibited significantly lower hysteresis throughout fatigue testing, which indicates greater elasticity in SDFT fascicles. Average stresses applied were comparable between tendon types (Table 1). However, it is difficult to directly relate diameter with material properties in a complex composite tissue such as tendon, owing to the inhomogeneous composition of the cross section. Therefore it is also relevant to compare the fatigue load applied, which was on average 1.1 N greater in CDET fascicles. It is possible that the higher applied loads in CDET fascicles may have accounted for some of the difference in fatigue properties observed between tendon types, but is extremely unlikely to result in the 20-fold difference in number of cycles to failure between the SDFT and CDET. Interestingly, fascicles from the SDFT exhibited less elongation initially, but were able to withstand greater elongation prior to failure than those from the CDET. However, if elongation in SDFT fascicles is calculated at the average cycle number at which CDET fascicles fail, this elongation is considerably less than observed in CDET fascicles (0.42 mm vs. 1.42 mm), suggesting that the greater elongation seen in the SDFT fascicles at failure is as a consequence of the larger number of loading cycles resisted prior to failure.

Both loading and unloading stiffness were significantly higher in the 10 cycles prior to failure in the CDET than in the SDFT, indicating greater alterations in the mechanical properties of CDET fascicles with fatigue loading. No previous studies have determined the fatigue resistance of the SDFT and CDET as a whole, however it has been shown that energy storing tendons exhibit greater fatigue resistance than positional tendons [6], [7]. Indeed, the time to rupture for highly stressed wallaby flexor tendons is approximately 10–20 times greater than that for extensor tendons, which experience much lower stresses in life [7]. In the current study, we applied a maximum load equivalent to 50% of the predicted failure force. The energy storing SDFT is predicted to experience loads of up to 80% of failure force *in vivo* during intense exercise [3], [24]. By contrast, maximum forces in the positional CDET are unlikely to exceed 25% of the tendon’s failure force [3], [24]. It has not been established how much load an individual fascicle may experience *in vivo*, but it is likely that the forces applied in the current study far exceed those experienced *in vivo* by the CDET, which may explain the extremely low fatigue resistance of the fascicles from this positional tendon. It has previously been established that loading of tendons to the stress they experience ‘in life’ results in a similar time to failure for all tendon types [7], [24]. It is not possible to perform these type of experiments at the micromechanical level, as the stress in life experienced by fascicles and IFM in functionally distinct tendons is yet to be determined.

Previous studies have demonstrated how fatigue damage accumulates in tendon and how this affects mechanical properties. Fung et al. [25] characterised the mechanical and structural alterations in the rat patellar tendon throughout fatigue loading, demonstrating that collagen fibre kinking was observed during the early stages of fatigue. With high levels of fatigue loading, damage was characterised by severe matrix disruption, poor fibre alignment, and widening of interfibre space [25]. This was associated with increased hysteresis and decreased stiffness, similar to that observed in the current study.

The micromechanical response to fatigue loading of isolated fascicles has also been characterised previously, with fibre kinking and matrix disruption observed, similar to that seen in whole tendons [19], [26]. This occurs even when relatively low stresses are applied [27]. The superior fatigue resistance of fascicles from the energy storing SDFT are likely due to specialisations that have been observed at the microstructural level. Our previous studies have demonstrated that fascicles in the SDFT have a helical substructure, allowing them to act as springs [28]. This helix is absent in CDET fascicles, in which extension occurs due to fibre sliding. This is associated with greater hysteresis and a lower ability to recover post-loading [28]. Indeed, it has been demonstrated that the helix substructure is lost in fascicles from aged SDFTs, and this is accompanied by a decrease in fatigue resistance [29].

In the SDFT, there were significant correlations between initial fascicle elongation, hysteresis, change in loading stiffness and fatigue resistance. However, these correlations were not present in the CDET, suggesting that the mechanisms of fatigue in each tendon type are fundamentally different. Previous studies of the fatigue response of the rat patellar tendon did not identify any correlation between elongation and hysteresis, but showed that hysteresis was correlated with the change in loading stiffness [30].

It is clear that the SDFT consists of highly specialised subunits that allow it to fulfil its energy storing function and resist high, repetitive stresses and strains. The equine SDFT has a function analogous to that of the human Achilles, and there is also a remarkably similar injury risk and aetiology between the two tendons [16], [17], therefore it seems logical to hypothesise that fascicles and IFM in the human Achilles tendon would show similarly high levels of fatigue resistance. However, anatomical differences exist between the tendons, and therefore further studies are required to determine the fatigue response of tendon subunits in the human Achilles.

## 5 Conclusion

This is the first study to assess the fatigue resistance of the tendon IFM, demonstrating that this structure has the ability to resist a significant amount of cyclic loading, both in the energy storing SDFT and positional CDET. Further, we have shown that both the IFM and fascicles in the energy storing SDFT are more fatigue resistant than those in the positional CDET, exhibiting less hysteresis and resisting a greater number of cycles prior to failure. These data suggest that both fascicles and IFM in the energy storing SDFT exhibit compositional and structural specialisations that likely contribute to superior fatigue resistance in the tendon as a whole. These findings provide important advances to further understand structure-function relationships within tendon.

## Figures and Tables

**Table 1:** Fatigue properties of fascicles and IFM from the SDFT and CDET. Data are displayed as mean ± SD. Significant differences between tendon types identified by: *^a^*p < 0.05; *^b^*p < 0.01; *^c^*p < 0.001.

	Fascicles		Interfascicular matrix	
SDFT	CDET	SDFT	CDET
Diameter (mm)	0.33 ± 0.14	0.37 ± 0.09*^b^*	–	–

Load applied (N)	3.68 ± 1.46	4.80 ± 1.88	1.15 ± 0.85	1.29 ± 1.28

Stress applied (MPa)	52.81 ± 28.62	47.99 ± 22.26	–	–

Number of cycles to failure	2709 ± 4819	139 ± 157*^c^*	921 ± 1947	215 ± 145*^b^*

Gradient of maximum creep curve	0.0014 ± 0.0023	0.013 ± 0.012*^c^*	0.0029 ± 0.0039	0.0082 ± 0.0046*^b^*

Gradient of minimum creep curve	0.00059 ± 0.00099	0.0054 ± 0.0091*^c^*	0.0012 ± 0.0014	0.0039 ± 0.0031

Hysteresis (%): Cycle 1–10	26.26 ± 7.31	34.05 ± 7.92*^b^*	30.57 ± 12.24	41.57 ± 12.48
Cycle 11–19	14.91 ± 4.23	23.77 ± 6.60*^c^*	20.38 ± 7.73	29.06 ± 12.74
Mid test cycles	12.22 ± 3.70	21.48 ± 7.56*^c^*	15.08 ± 6.69	22.06 ± 7.63*^b^*
Last 10 cycles	39.93 ± 11.05	53.12 ± 15.86*^c^*	53.32 ± 10.06	69.44 ± 11.38*^b^*

Loading stiffness (N/mm): Cycle 1	5.21 ± 1.75	6.23 ± 1.97*^a^*	2.69 ± 1.23	2.82 ± 1.37
Cycle 10	5.18 ± 1.82	5.36 ± 2.00	2.50 ± 1.29	2.03 ± 1.25*^a^*
Mid test cycle	4.23 ± 1.51	5.36 ± 1.33	2.54 ± 1.05	1.90 ± 1.04
10 cycles before failure	2.85 ± 1.10	4.51 ± 1.27*^c^*	2.06 ± 0.74	1.43 ± 0.77
Last cycle	2.50 ± 0.88	3.68 ± 1.36*^c^*	1.57 ± 0.60	1.32 ± 0.86

Unloading stiffness (N/mm): Cycle 1	8.56 ± 3.43	10.54 ± 3.25	4.73 ± 1.64	5.50 ± 2.22
Cycle 10	7.38 ± 2.90	8.23 ± 2.76	4.08 ± 1.79	3.70 ± 2.05
Mid test cycle	6.46 ± 2.44	8.39 ± 1.70	3.86 ± 1.44	3.25 ± 1.59
10 cycles before failure	5.35 ± 1.84	7.83 ± 2.18*^a^*	3.62 ± 1.00	2.70 ± 1.43
Last cycle	5.89 ± 2.49	9.06 ± 4.46*^a^*	3.64 ± 1.26	4.05 ± 2.19

Elongation (mm): Cycles 1–10	0.063 ± 0.042	0.29 ± 0.25*^c^*	–	–
Cycles 1-failure	1.79 ± 0.49	1.42 ± 0.41*^a^*	2.18 ± 1.82	1.32 ± 0.72

**Table 2:** Correlations between initial mechanical testing parameters (hysteresis, elongation at cycle 10 and increase in loading stiffness) and the number of cycles to failure in fascicles from the SDFT. There were no significant correlations between any of these parameters in the CDET. NS = not significant.

	Hysteresis (%)	Elongation (mm)	Cycles to failure
Hysteresis (%)	–	p = 0.047	p = 0.0072
r = 0.51	r = −0.68

Elongation (mm)	–	–	p = 0.037
	r = −0.55

Change in loading stiffness (%)	NS	p = 0.013	p = 0.006
r = −0.61	r = 0.73
